# Outcomes of Patients With ST-Segment Elevation Myocardial Infarction Admitted During COVID-19 Pandemic Lockdown in Germany – Results of a Single Center Prospective Cohort Study

**DOI:** 10.3389/fcvm.2021.638954

**Published:** 2021-04-20

**Authors:** Manuel Rattka, Lina Stuhler, Claudia Winsauer, Jens Dreyhaupt, Kevin Thiessen, Michael Baumhardt, Sinisa Markovic, Wolfgang Rottbauer, Armin Imhof

**Affiliations:** ^1^Clinic for Internal Medicine II, University Hospital Ulm - Medical Center, Ulm, Germany; ^2^Institute of Epidemiology and Medical Biometry, Ulm University, Ulm, Germany

**Keywords:** COVID-19, STEMI, myocardial infarction, lockdown, outcome, epidemiology, Germany

## Abstract

**Objective:** Since the outbreak of the COVID-19 pandemic, healthcare professionals reported declining numbers of patients admitted with ST-segment myocardial infarction (STEMI) associated with increased in-hospital morbidity and mortality. However, the effect of lockdown on outcomes of STEMI patients admitted during the COVID-19 crisis has not been prospectively evaluated.

**Methods:** A prospective, observational study on STEMI patients admitted to our tertiary care center during the COVID-19 pandemic was conducted. Outcomes of patients admitted during lockdown were compared to those patients admitted before and after pandemic-related lockdown.

**Results:** A total of 147 patients were enrolled in our study, including 57 patients in the pre-lockdown group (November 1, 2019 to March 20, 2020), 16 patients in the lockdown group (March 21 to April 19, 2020), and 74 patients in the post-lockdown group (April 20 to September 30, 2020). Patients admitted during lockdown had significantly longer time to first medical contact, longer door-to-needle-time, higher serum troponin T levels, worse left ventricular end-diastolic pressure, and higher need for circulatory support. After a median follow-up of 142 days, survival was significantly worse in STEMI patients of the lockdown group (log-rank: *p* = 0.0035).

**Conclusions:** This is the first prospective study on outcomes of STEMI patients admitted during public lockdown amid the COVID-19 pandemic. Our results suggest that lockdown might deteriorate outcomes of STEMI patients. Public health strategies to constrain spread of COVID-19, such as lockdown, have to be accompanied by distinct public instructions to ensure timely medical care in acute diseases such as STEMI.

## Introduction

Soon after the severe acute respiratory syndrome coronavirus 2 (SARS-CoV-2) spread globally, physicians warned about potential side effects of the COVID-19 pandemic compromising medical care (1, 2). It has been suggested that the pandemic keeps patients from seeking and receiving needed medical attention despite suffering from physical symptoms. Social containment measures (i.e., lockdown and stay-at-home orders), stress, and fear of COVID-19 may influence an individual's health behavior (3–5). Patients with ST-segment elevation myocardial infarction (STEMI) are an especially vulnerable population, as total ischemic time severely influences their outcome (6). There have been several reports on diminishing numbers of STEMI admissions during the outbreak in both epicenters and non-epicenters of the COVID-19 pandemic. This has been associated with significantly prolonged times from symptom onset to first medical contact (FMC) and increased in-hospital morbidity and mortality (7–11). However, the reasons underlying this phenomenon have rarely been assessed. The influence of factors such as lockdown, stress, and fear of COVID-19 on the patient's long-term outcome, has not yet been evaluated.

## Methods

### Study Design and Study Population

In this prospective, observational cohort study, we aimed for inclusion of all patients with STEMI admitted between March 21, 2020 and September 30, 2020. STEMI patients admitted between November 1, 2019 and March 20, 2020 were enrolled retrospectively.

Patients had to be ≥18 years old and give written informed consent to be eligible for inclusion. Diagnosis of STEMI was made according to contemporary guidelines and all STEMI patients underwent cardiac catheterization and subsequent percutaneous coronary intervention (PCI) immediately after admission as indicated by current recommendations (6). During the COVID-19 pandemic, all patients were treated with personal protection gear in the case of an unknown COVID-19 status. The study complies with the Declaration of Helsinki and was approved by the local ethics committee (number of application and positive vote 250/20). This study adheres to the STROBE statement (12).

### Data Collection

Demographic, clinical, laboratory, interventional, and in-hospital outcome data were extracted from our patient management system by two medical practitioners (CW and LS) and adjudicated by a third one (MR) in case of any kind of difference. Left ventricular systolic function at admissions was measured by cardiac ventriculography during cardiac catheterization and categorized as normal, mildly impaired, moderately impaired, or severely impaired, according to the expertise of the attending physician. Left ventricular systolic function at follow-up was assessed by automated echocardiographic quantification (outpatient visit: EPIQ 7, Koninklijke Philips N.V., Eindhoven, Netherlands; home visit: Butterfly IQ, Butterfly Network. Inc., Guilford, CT, USA).

### Clinical Follow-Up

Patients were scheduled for outpatient clinic visits (clinical assessment, 12-lead ECG, and echocardiography) after 1 month, 3 months and, then, at least every 3 months after discharge. If, for any reason, an outpatient clinic visit could not be realized, a home visit was offered to the patient.

### Laboratory Measurements

Blood samples were drawn at the time of hospital admission or at the outpatient clinic visits for measurements of high sensitivity cardiac troponin T (hsTnT), NT-pro BNP, and creatinine (ElectroChemiLumineszenz ImmunoAssay “ECLIA” Roche, Cobas 8000, Modul e801 and e601) as part of the clinical routine. In addition, every patient was tested for SARS- CoV-2 by throat swab test at admissions (Sigma-Virocult^®^ with 2 ml Virocult^®^ medium, Check Diagnostics GmbH, Germany) and analyzed by RT-PCR at the local Institute for Virology.

### Assessment of the Effect of Lockdown on STEMI Patients

Measures of social restriction in Germany came into effect on March 21, 2020 and public reopening was partly initiated on April 20, 2020. Consequently, patients admitted between November 1, 2019 and March 20, 2020 were classified as the “pre-lockdown” (pre-COVID-19) group, patients admitted between March 21 and April 19, 2020 were assigned to the “lockdown” group, and patients admitted between April 20 and September 30, 2020 to the “post-lockdown” group. Comparisons were made on patient characteristics, clinical data, and outcomes of patients of the lockdown group and the combined pre-/post-lockdown group. Outcomes were heart failure symptoms as measured by NYHA class, serum levels of cardiac biomarkers, left ventricular ejection fraction, and survival. Additionally, baseline characteristics, laboratory parameters, in-hospital clinical characteristics and time to FMC were assessed and compared between the groups.

### Assessment of the Effect of Stress and Fear of COVID-19 on STEMI Patients Admitted During the COVID-19 Pandemic

To assess the level of stress and fear of COVID-19 at baseline in STEMI patients admitted during the pandemic, we utilized well-established questionnaires. The COVID Stress Scales (CSS) were used to assess COVID-19 related distress and the Fear of COVID-19 Scale (FCV-19S) was implemented to measure COVID-19 related fear (13, 14).

### Statistical Analysis

Continuous variables were described as mean ± standard deviation or median together with interquartile range (IQR), as appropriate. Categorical variables were described as absolute and relative frequencies, respectively. Group comparison (lockdown vs. pre-/post-lockdown combined) of continuous variables was performed by two-sample *t*-test or Wilcoxon rank sum test as appropriate. Group comparison (pre-lockdown vs. lockdown vs. post-lockdown) of continuous variables was performed by one-way ANOVA or Kruskal-Wallis test as appropriate. The chi^2^ test or Fisher's exact test was used for group comparison of categorical variables. The Fisher's exact test was used if >20% of cells of the table contain expected values of <5, as appropriate. Otherwise the chi-squared test was used. The Kaplan-Meier estimator was used to assess the time to event and groups were compared using the log-rank test. Logistic regression analysis was done to investigate potential predictors on delayed presentation. Association of outcomes and total sums of both CSS and FCV-19S were assessed by scatter plots and either point-biserial correlation coefficient (in the case of dichotomous variables) or Spearman rank correlation coefficient (in the case of continuous variables).

Statistical analysis was performed by SAS version 9.4 under Windows. A two-sided *p*-value of <0.05 was considered statistically significant. Due to the explorative nature of this study, all results from statistical tests have to be interpreted as hypothesis generating. An adjustment for multiple testing was not done.

## Results

### Patient Characteristics

From March 21, 2020, when measures of social restrictions were implemented for the first time during the COVID-19 pandemic in Germany, until the end of our inclusion period on September 31, 2020, 90 patients with STEMI had been admitted to our tertiary care center. Amongst those, 16 patients had been admitted during the lockdown period (March 21, 2020 to April 19, 2020; “lockdown group”) and 74 patients in the post-lockdown period (April 20, 2020 to September 30, 2020; “post-lockdown group”). Furthermore, characteristics of 57 STEMI patients admitted before the COVID-19 pandemic (“pre-lockdown group”) were assessed. For main analyses, the “pre-lockdown group” and the “post-lockdown group” were combined (“pre-/post-lockdown group”). In total, the mean age was 64 ± 13 years with 76% (112 out of 147 patients) being male. There were no significant differences in baseline characteristics between groups. No patients tested positive for SARS-CoV-2 virus during hospitalization. No patient was lost to follow-up. Detailed baseline characteristics are shown in Table 1 and Supplementary Table 1.

**Table 1:** Patient characteristics at baseline.

	**Total**	**Lockdown**	**Pre-/Post-Lockdown**	***p*-value**
	***n* = 147**	***n* = 16**	***n* = 131**	
Age	64 ± 13	69 ± 12	64 ± 14	0.1519^*^
Sex (male)	112 (76)	12 (75)	100 (76)	1.0000^§^
Arterial hypertension	89 (61)	11 (69)	78 (60)	0.4768^§§^
Diabetes	39 (27)	3 (19)	36 (27)	0.5608^§^
Family history	35 (24)	3 (19)	32 (24)	0.7627^§^
Smoking	71 (48)	8 (50)	63 (48)	0.8853^§§^
Obesity	21 (14)	2 (13)	19 (15)	1.0000^§^
TIA/stroke	8 (5)	2 (13)	6 (5)	0.2109^§^
OSAS	7 (5)	1 (6)	6 (5)	0.5616^§^
COPD	2 (3)	1 (6)	4 (3)	0.4427^§^
CKD	35 (24)	5 (31)	30 (23)	0.5345^§^
FCV-19S questionnaire (score)	14 (9, 17)	12 (9, 17)	14 (9, 17)	0.8976^**^
CSS questionnaire (score)	38 (25, 70)	31 (13, 50]	39 (27, 71)	0.2018^**^

*TIA, transient ischemic attack; OSAS, obstructive sleep apnea syndrome; COPD, chronic obstructive pulmonary disease; CKD, chronic kidney disease; FCV-19S, Fear of COVID-19 Scale; CSS, COVID-19 Stress Scales*.

* *two-sample t-test*.

** *Wilcoxon rank sum test*.

§ *Fisher's exact test*.

§§ *chi^2^ test*.

### Clinical Characteristics at Admission

To assess the effect of lockdown on STEMI patients admitted to hospital during the COVID-19 outbreak, clinical characteristics were assessed and compared to patients admitted before the outbreak and those admitted after measures of social restrictions had been lifted. Remarkably, a significantly higher rate of patients in the lockdown period reported that they intentionally did not go to the hospital or inform the emergency medical services immediately after the onset of symptoms (pre-/post-lockdown: 39 out of 120 patients (33%), lockdown: 11 out of 13 patients (85%); *p* = 0.0004). Likewise, 46% of patients in the lockdown group acknowledged that the time from symptom onset to FMC was longer than 24 h compared to 11% of patients in the pre-/post-lockdown group. Overall, time to FMC (in hours) was significantly prolonged in the lockdown group [pre-/post-lockdown: 2.0 (0.3, 16.0), lockdown: 11.0 (2.0, 144.0); *p* = 0.0193]. Additionally, door-to-needle time (in minutes) was significantly prolonged in patients admitted during lockdown [pre-/post-lockdown: 46 (28, 74); lockdown: 83 (59, 117); *p* = 0.0277]. Interestingly, patients in the lockdown group were more symptomatic at admission, as measured by NYHA class. However, there was no significant difference for measurements of vital signs at admission. Evaluation of laboratory parameters at admissions revealed that patients admitted due to STEMI during lockdown had significantly higher serum troponin T levels compared to those admitted before and after the pandemic lockdown [pre-/post-lockdown: 244 (53, 1124) ng/L, lockdown: 746 (292, 3899) ng/L; *p* = 0.0105]. Additionally, measures for NT-pro BNP and creatinine showed no significant difference. However, mean left ventricular end diastolic pressure (LVEDP) was significantly higher in the lockdown group compared to the pre-/post-lockdown group [pre-/post-lockdown: 24 (17, 29) mmHg, lockdown: 34 (27, 36) mmHg; *p* = 0.0116]. Lastly, STEMI patients admitted during lockdown had significantly higher need for circulatory support than those admitted before after the lockdown period [pre-/post lockdown: 18 out of 122 patients (15%), lockdown: nine out of 16 patients (56%), *p* = 0.0005]. Clinical characteristics at admission are summarized in Table 2 and Supplementary Table 2.

**Table 2:** Clinical characteristics at baseline.

	**Total**	**Lockdown**	**Pre-/Post-Lockdown**	***p*-value**
	***n* = 147**	***n* = 16**	***n* = 131**	
**NYHA class**				
I	34 (27)	1 (8)	33 (29)	**0.0087**^**§**^
II	25 (20)	0 (0)	25 (22)	
III	10 (8)	3 (23)	7 (6)	
IV	57 (45)	9 (69)	48 (42)	
**Delayed presentation**				
Yes	50 (38)	11 (85)	39 (33)	**0.0004**^**§**^
No	83 (62)	2 (15)	81 (68)	
**Time to FMC**				
Immediately	60 (45)	2 (15)	58 (49)	**0.0032**^**§**^
≤ 3 h	27 (20)	2 (15)	25 (21)	
≤ 12 h	14 (11)	3 (23)	11 (9)	
≤ 24 h	12 (9)	0 (0)	12 (10)	
> 24 h	19 (14)	6 (46)	13 (11)	
Time to FMC (hours)	2.0 (0.3, 24.0)	11.0 (2.0, 144.0)	2.0 (0.3, 16.0)	0.0193^**^
Systolic bp (mmHg)	117 ± 28	116 ± 29	117 ± 29	0.9009^*^
Diastolic bp (mmHg)	67 ± 20	76 ± 19	65 ± 19	0.0579^*^
Troponin T (ng/L)	318 (63, 1,301)	746 (292, 3,899)	244 (53, 1,124)	0.0105^**^
NT-pro BNP (pg/ml)	354 (91, 1,879)	1,120 (237, 6,459)	331 (83, 1,712)	0.0717^**^
Creatinine (μmol/L)	84 (71, 110)	86 (74, 115)	84 (71, 109)	0.6503^**^
**Laevocardiography**				
Normal	4 (3)	0 (0)	4 (3)	0.2620^§^
Mildly reduced	31 (23)	4 (27)	27 (22)	
Moderately reduced	55 (40)	3 (20)	52 (43)	
Severely reduced	46 (34)	8 (53)	38 (31)	
LVEDP (mmHg)	26 (17, 32)	34 (27, 36)	24 (17, 29)	0.0116^**^
Door-to-needle-time (min)	54 (28, 80)	83 (59, 117)	46 (28, 74)	0.0277^**^
**Culprit lesion**				
LAD	67 (49)	11 (79)	56 (46)	0.0815^§^
LCX	19 (14)	1 (7)	18 (15)	
RCA	51 (37)	2 (14)	49 (40)	
**Circulatory support**				
Yes	27 (20)	9 (56)	18 (15)	**0.0005**^**§**^
No	111 (80)	7 (44)	104 (85)	
Time at hospital (days)	4 (3, 6)	5 (2, 6)	4 (3, 6)	0.9445^**^

*FMC, first medical contact; BNP, brain natriuretic peptide; bp, blood pressure; LVED, left ventricular end diastolic pressure; LAD, left anterior descending; LCX, left circumflex artery; RCA, right coronary artery*.

* *two-sample t-test*.

** *Wilcoxon rank sum test*.

§ *Fisher's exact test*.

*^§§^ chi^2^ test*.

*Boldface denotes significance of p-values*.

### Clinical Outcomes

**Figure 1:**
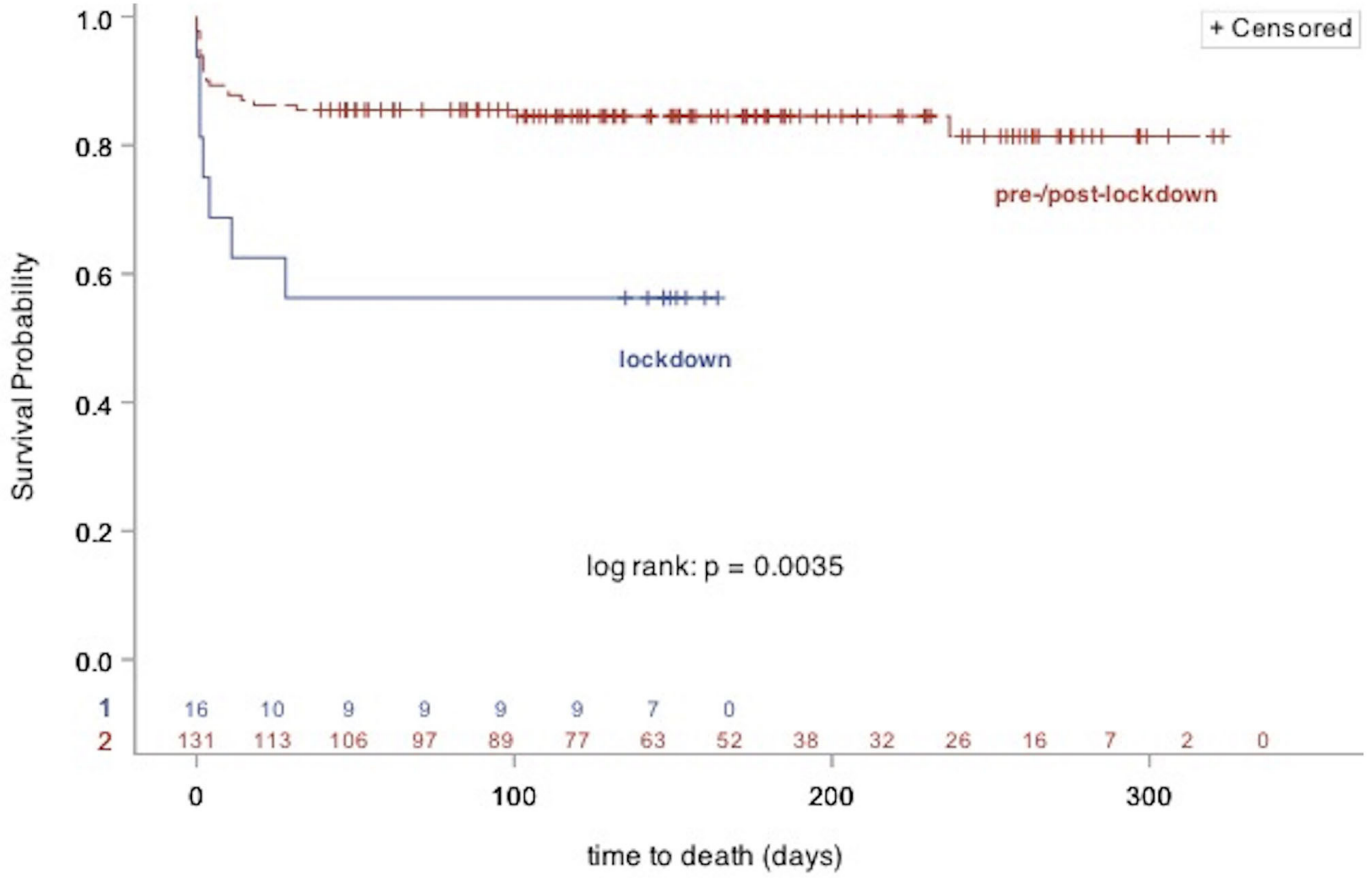
Kaplan Meier plot showing survival of STEMI patients admitted during (lockdown group), and outside of COVID-19 associated lockdown (pre-/post-lockdown group).

Patients included in our analysis had a median follow-up time of 142 days. Intriguingly, a comparison of survival time of patients of the lockdown group and patients of the pre-/post-lockdown group showed that STEMI patients admitted during lockdown had a significantly lower survival (confirmed deaths; pre-/post-lockdown: 21 out of 131 patients; lockdown: 7 out of 16 patients; log-rank test: *p* = 0.0035; Figure 1). This was associated with a higher rate of patients in the lockdown group (30%) reporting the presence of heart failure symptoms at rest compared to the pre-/post-lockdown period (11%). However, the overall difference in NYHA-class between both groups was only a non-significant result (*p* = 0.1367; Table 3). Analysis of laboratory measures at follow-up showed no significant difference. Clinical outcomes are shown in Table 3 and Supplementary Table 3.

**Table 3:** Patient characteristics at follow up.

	**Total**	**Lockdown**	**Pre-/Post-Lockdown**	***p*-value**
	***n* = 147**	***n* = 16**	***n* = 131**	
**NYHA class**				
I	46 (45)	5 (50)	41 (44)	0.1367^§^
II	35 (34)	1 (10)	34 (37)	
III	9 (9)	1 (10)	8 (9)	
IV	13 (13)	3 (30)	10 (11)	
LVEF	53 (45, 60)	47 (35, 63)	53 (45, 60)	0.4327^**^
Troponin T (ng/L)	19 (10, 39)	26 (19, 81)	17 (10, 33)	0.1300^**^
NT-pro BNP (pg/ml)	483 (187, 1,092)	1,014 (187, 3,559)	483 (195, 964)	0.4976^**^
Creatinine (μmol/L)	81 (74, 93)	83 (74, 131)	80 (74, 93)	0.5377^**^

*NYHA, New York Heart Association; LVEF, left ventricular ejection fraction; BNP, brain natriuretic peptide*.

** *Wilcoxon rank sum test*.

§ *Fisher's exact test*.

### Effect of Stress of COVID-19 and Fear of COVID-19 on Outcomes

Since the association of fear of COVID-19 and outcomes of STEMI patients has not been comprehensively evaluated so far, we assessed the level of stress and fear of COVID-19 by two well-established questionnaires (FCV-19S and CSS). Patients in the lockdown period had a median FCV-19S Score of 12 (9, 17) and CSS score of 31 (13, 50), compared to a FCV-19S score of 14 (9, 17) (*p* = 0.8976), and CSS Score of 39 (27, 71) (*p* = 0.2018) in the pre-/post-lockdown group (Table 1). Association analysis of total test scores with baseline and follow-up parameters showed a significant relationship between the total CCS score and left ventricular contractile function as assessed by laevocardiography. However, no association between the total FCS-19V score and laevocardiography at admission could be demonstrated. There was no relationship between anxiety of COVID-19 and other parameters (Supplementary Table 4).

### Predictors of Intentionally Delayed Presentation

To identify predictors of delayed presentation, we performed both univariate as well as multiple logistic regression analysis of the parameters that potentially keep STEMI patients from seeking timely medical care amid the COVID-19 pandemic. After multiple analysis, only “admission during lockdown” remained significantly associated with intentionally delayed presentation (Table 4).

**Table 4:** Identification of predictors of intentionally delayed presentation.

	**Multiple analysis**		
**Variables**	**Odds Ratio (OR)**	**95% CI of OR**	***p*-value**
Age	0.965	0.924–1.008	0.1133
Sex (female)	2.187	0.567–8.437	0.2558
Admission during lockdown	16.393	1.692–166.67	**0.0159**
FCV-19S questionnaire (score)	1.083	0.939–1.249	0.2760
CSS questionnaire (score)	0.986	0.952–1.021	0.4280

*FCV-19S, Fear of COVID-19 Scale; CSS, COVID-19 Stress Scales. Boldface denotes significance of p-values*.

## Discussion

To the best of our knowledge, this is the first study prospectively evaluating the outcome of STEMI patients admitted during the COVID-19 pandemic caused lockdown, which also analyzes the effects of stress and fear of COVID-19 on patient outcomes. We found that patients with STEMI admitted during the lockdown period to our tertiary center showed lower survival compared to both those admitted before the COVID-19 pandemic and after measures of social restriction have been partly lifted. This was associated with a longer time from symptom onset to FMC and a prolonged door-to-needle time. Additionally, patients in the lockdown group had significantly higher serum troponin T levels, a worse left ventricular end-diastolic pressure, and a higher need of circulatory support at admission.

### Effect of Fear of COVID-19 and Stress of COVID-19 on STEMI Patients

Observations since the beginning of the COVID-19 crisis have suggested that various factors, such as altruistic behavior, information by the media, and especially fear of contagion with SARS-CoV-2 in hospital, contributed to reduced admissions of patients with acute myocardial infarction and prolonged times from symptom onset to FMC (4, 15). It has been reported, that patients who avoided an emergency room visit timely because they feared getting infected with SARS-CoV-2 in hospital, suffered catastrophic complications such as ventricular septal defect, which has become rare due to steadily improving medical care (16). As fear displays one characteristic of infectious disease and is associated with its transmission rate, morbidity, and mortality, Ahorsu et al. developed and validated a 7-item scale (Fear of COVID-19 Scale, FCV-19S) assessing the level of fear of SARS-CoV-2 (14). As there is currently no systematic study on the effect of fear on patients with myocardial infarction, we employed the FCV-19S to estimate if the extent of fear of COVID-19 is associated with worsened outcomes in STEMI patients. To do so, we conducted association analysis of the scores of the FCV-19S, and relevant clinical patient characteristics. Our results indicate that overall fear of COVID-19 is not related to adverse outcomes in STEMI patients. To substantiate this finding, we applied the COVID Stress Scales, a 36-item scale developed by Taylor et al. to better understand and assess COVID-19-related stress and anxiety (13). Besides a single relationship between the total CSS score and left ventricular systolic contractility as assessed by laevocardiography, we could not demonstrate an association between the totaled item scores and outcomes of STEMI patients. Therefore, fear of COVID-19 was not associated with lockdown, higher measures of cardiac biomarkers, outcomes at follow-up, and prolonged times from symptom onset to FMC. Since our study population represents a region which has been rather spared from overwhelming infection rates in the early phase of the pandemic, these results might deviate if STEMI patients in epicenters of the pandemic are interviewed.

### Effect of Lockdown on STEMI Patients

Soon after SARS-CoV-2 surfaced around the world, several countermeasures were initiated to contain further spread as much as possible. Drastic measures of social distancing and public lockdown were implemented, among other policies. In Germany, public facilities were closed, sporting events were canceled, and the physical contact of more than two persons outside of families was prohibited (9). Depending on the region, even curfews were enforced to minimize the inter-personal contact. For patients suffering from acute myocardial infarction, it has been suggested that measures of public lockdown might interfere with timely and adequate medical care (1, 2, 4, 10). It has been observed that the implementation of regional lockdown was associated with a significant decline in admission numbers of STEMI patients compared to times before the pandemic (10, 17). Furthermore, a concomitant increase of patient-related as well as system-related delay times, as measured by the time from symptom onset to FMC and door-to-balloon time, has been registered (18). However, it is difficult to distinguish if these findings were related specifically to the lockdown or to the COVID-19 crisis as a whole. To date, there are only a few retrospective cohort and register studies available with data on STEMI patients admitted during and after regional lockdown (19, 20). While these studies confirm the decrease in incidence during lockdown period, there is a lack of information regarding delay times, mortality and survival (19, 20). To compensate for this issue, we prospectively assessed and compared survival of STEMI patients admitted during the COVID-19 pandemic and outside of lockdown. Intriguingly, we found that the lockdown group had a significantly lower survival. This might be attributed to our finding that during lockdown, patients were admitted in worse condition. This is substantiated by (1) worse symptoms as measured by NYHA-class, (2) significantly increased serum troponin T levels, (3) a significantly higher LVEDP, and (4) significantly higher need for circulatory support in the lockdown-group. This could be related to a significantly prolonged time from symptom onset to FMC during lockdown, which is known to be associated with larger infarct size and infarct transmurality (21). Additionally, we observed that the door-to-needle time was significantly prolonged in the lockdown-group, too. Evidently, this is related to the indispensable adaptation of emergency processes, such as employment of personal protective gear, to mitigate the risk of getting infected with SARS-CoV-2 (22). Nevertheless, it remains possible that the increase in system-related delay time, as measured by door-to-needle time, contributed to the worse outcome of STEMI patients admitted during lockdown. Assessment of other outcomes did not show differences, which might be related to the higher number of deceased patients in the lockdown group, who, therefore, did not receive a follow-up visit.

Moreover, by multiple logistic regression analysis, we show that amongst several aforementioned factors that potentially keep STEMI patients from seeking timely medical attention despite experiencing ischemic symptoms, that lockdown (not stress or fear of COVID-19) was significantly associated with an intentionally delayed presentation. These findings substantiate our hypothesis that measures of social distancing such as lockdown adversely affect the health behavior and outcomes of STEMI patients.

As a consequence, public lockdown appears to considerably deteriorate the prognosis of patients suffering from myocardial ischemia. In the presence of the currently rising incidence of SARS-CoV-2 virus infections worldwide and imminent recurrence of lockdown measures, public health policy has to carefully decide on the extent of social policies to avoid potential excess morbidity and mortality. Implementation of lockdown measures have to be accompanied by distinct public instructions on how to act in health emergencies such as STEMI and others.

### Limitations

As this is a prospective, observational explorative study on the outcomes of STEMI patients admitted during and outside of social lockdown related to the COVID-19 pandemic, it inherently has limitations. Since this is a study from a single center, only a limited number of patients could be included. Due to the explorative character of this study, our results have to be interpreted as hypothesis generating. Studies reporting the outcomes a larger number of participants, which might be achievable by a multi-center design or a prolonged time to select cases, are of the essence to verify our results and to further investigate the drivers of increased mortality (e.g., by pathway analysis). Nonetheless, these results are the first prospective data on the outcomes of STEMI patients admitted during, before and after lockdown, which reveal a significant decrease in survival during lockdown. For further analysis, the raw data underlying our analyses are available upon publication.

## Conclusion

This is the first prospective study comparing the outcomes of STEMI patients admitted during lockdown, to outcomes of patients admitted before and after public lockdown in a non-COVID-19 epicenter. Our results suggest that enforced lockdown is associated with reduced survival of STEMI patients, which supposedly is related to prolonged patients delay times. Patient related factors such as the fear of getting infected in the hospital or stress factors related to COVID-19 seem to have less impact on outcomes among these patients. Public health care strategies to constrain SARS-CoV-2 or other pandemics at present and in future including public lockdown measures have to assure timely medical treatment beyond COVID-19. Implementation of lockdown measures should be accompanied by distinct public instructions on how to act in acute life-threatening diseases such as STEMI and others.

## Data Availability Statement

The raw data supporting the conclusions of this article will be made available by the authors, without undue reservation. Mendeley Data can be found with 10.17632/8pzrkkjrz3.1.

## Ethics Statement

The studies involving human participants were reviewed and approved by Ulm University Ethics Committee. The patients/participants provided their written informed consent to participate in this study.

## Author Contributions

MR and AI had the idea for and designed the study and had full access to all data and take responsibility for the integrity of the data and the accuracy of the data analysis. MR, CW, and LS collected the data. JD performed the statistical analysis. MR and KT mainly wrote the manuscript with support from AI, CW, LS, SM, and MB. MR, AI, and WR were mainly responsible for the interpretation of the data. AI and WR supervised the project. All authors contributed to the article and approved the submitted version.

## Conflict of Interest

The authors declare that the research was conducted in the absence of any commercial or financial relationships that could be construed as a potential conflict of interest.
