# Supplementary material for: Outcomes of Patients With ST-Segment Elevation Myocardial Infarction Admitted During COVID-19 Pandemic Lockdown in Germany – Results of a Single Center Prospective Cohort Study
Source: Front Cardiovasc Med. 2021 Apr 20;8:638954. doi: 10.3389/fcvm.2021.638954 (PMC8093511; doi:10.3389/fcvm.2021.638954)
Supplement: Supplementary file 1 [file Data_Sheet_1.docx]

**Supplementary Material:**

| Supplementary Table 1. Patient characteristics at baseline | | | | | |
| --- | --- | --- | --- | --- | --- |
|  | Total | Pre-lockdown | Lockdown | Post-lockdown | p value |
|  | n = 147 | n = 57 | n = 16 | n = 74 |  |
| Age | 64 ± 13 | 64 ± 13 | 69 ± 12 | 64 ± 14 | 0.3575^*^ |
| Sex (male) | 112 (76) | 47 (82) | 12 (75) | 53 (72) | 0.3504^§§^ |
| Arterial hypertension | 89 (61) | 36 (63) | 11 (69) | 42 (57) | 0.5891^§§^ |
| Diabetes | 39 (27) | 15 (26) | 3 (19) | 21 (28) | 0.7306^§§^ |
| Family history | 35 (24) | 18 (32) | 3 (19) | 14 (19) | 0.2124^§§^ |
| Smoking | 71 (48) | 28 (49) | 8 (50) | 35 (47) | 0.9686^§§^ |
| Obesity | 21 (14) | 7 (12) | 2 (13) | 12 (16) | 0.7969^§§^ |
| TIA / stroke | 8 (5) | 5 (9) | 2 (13) | 1 (1) | **0.0495**^§^ |
| OSAS | 7 (5) | 1 (2) | 1 (6) | 5 (7) | 0.3645^§^ |
| COPD | 2 (3) | 3 (5) | 1 (6) | 1 (1) | 0.2956^§^ |
| CKD | 35 (24) | 12 (21) | 5 (31) | 18 (24) | 0.6915^§§^ |
| FCV-19S questionnaire (score) | 14 [9, 17] | N/A | 12 [9, 17] | 14 [9, 17] | 0.8976^*^ |
| CSS questionnaire (score) | 38 [25, 70] | N/A | 31 [13, 50] | 39 [27, 71] | 0.2018^*^ |
| TIA, transient ischemic attack; OSAS, obstructive sleep apnea syndrome; COPD, chronic obstructive pulmonary disease; CKD, chronic kidney disease; FCV-19S, Fear of COVID-19 Scale; CSS, COVID-19 Stress Scales; N/A, not available  ^*^ one-way ANOVA  ^§^ Fisher’s exact test  ^§§^ chi^2^ test | | | | | |

| Supplementary Table 2. Clinical characteristics at baseline | | | | | |
| --- | --- | --- | --- | --- | --- |
|  | Total | Pre-lockdown | Lockdown | Post-lockdown | p value |
|  | n = 147 | n = 57 | n = 16 | n = 74 |  |
| NYHA class  I  II  III  IV | 34 (27)  25 (20)  10 (8)  57 (45) | 25 (52)  18 (38)  3 (6)  2 (4) | 1 (8)  0 (0)  3 (23)  9 (69) | 8 (12)  7 (11)  4 (6)  46 (71) | **<0.0001^§^** |
| delayed presentation  yes  no | 50 (38)  83 (62) | 17 (34)  33 (66) | 11 (85)  2 (15) | 22 (31)  48 (69) | **0.0011**^§§^ |
| Time to FMC  Immediately  ≤ 3 hours  ≤ 12 hours  ≤ 24 hours  > 24 hours | 60 (45)  27 (20)  14 (11)  12 (9)  19 (14) | 21 (43)  11 (22)  8 (16)  5 (10)  4 (8) | 2 (15)  2 (15)  3 (23)  0 (0)  6 (46) | 37 (53)  14 (20)  3 (4)  7 (10)  9 (13) | **0.0083**^§^ |
| Time to FMC (hours) | 2.0 [0.3, 24] | 2.5 [0.3, 9.0] | 11.0 [2.0, 144.0] | 1.0 [0.3, 24.0] | **0.0487**^**^ |
| Systolic bp (mmHg) | 117 ± 28 | 112 ± 35 | 116 ± 29 | 119 ± 27 | 0.7706^*^ |
| Diastolic bp (mmHg) | 67 ± 20 | 61 ± 19 | 76 ± 19 | 66 ± 20 | 0.1088^*^ |
| Troponin T (ng/L) | 318 [63, 1301] | 266 [65, 1124] | 746 [292, 3899] | 193 [34, 1115] | **0.0330**^**^ |
| NT-pro BNP (pg/ml) | 354 [91, 1879] | 354 [74, 1489] | 1120 [237, 6459] | 327 [102, 1879] | 0.1899^**^ |
| Creatinine (µmol/L) | 84 [71, 110] | 87 [72, 111] | 86 [74, 115] | 84 [70, 102] | 0.5366^**^ |
| Laevocardiography  Normal  Mildly reduced  Moderately reduced  Severely reduced | 4 (3)  31 (23)  55 (40)  46 (34) | 2 (4)  12 (23)  19 (37)  19 (37) | 0 (0)  4 (27)  3 (20)  8 (53) | 2 (3)  15 (22)  33 (48)  19 (28) | 0.4140^§^ |
| LVEDP (mmHg) | 26 [17, 32] | 27 [20, 35] | 34 [27, 36] | 24 [17, 28] | **0.0104**^**^ |
| Door-to-needle-time (min) | 54 [28, 80] | 47 [22, 89] | 83 [59, 117] | 45 [29, 71] | 0.0818^**^ |
| Culprit lesion  LAD  LCX  RCA | 67 (49)  19 (14)  51 (37) | 29 (56)  5 (10)  18 (35) | 11 (79)  1 (7)  2 (14) | 27 (38)  13 (18)  31 (44) | **0.0490**^§^ |
| Circulatory support  yes  no | 27 (20)  111 (80) | 7 (13)  47 (87) | 9 (56)  7 (44) | 11 (16)  57 (84) | **0.0004**^§§^ |
| Time at hospital (days) | 4 [3, 6] | 4 [3, 5] | 5 [2, 6] | 5 [4, 6] | 0.1808^**^ |
| FMC, first medical contact; BNP, brain natriuretic peptide; bp, blood pressure; LVED, left ventricular end diastolic pressure; LAD: left anterior descending; LCX: left circumflex artery; RCA: right coronary artery  ^*^ one-way ANOVA  ^**^ Kruskal-Wallis test  ^§^ Fisher’s exact test  ^§§^ chi^2^ test | | | | | |

| Supplementary Table 3. Patient characteristics at follow-up |
| --- |

|  | Total | Pre-lockdown | Lockdown | Post-lockdown | p value |
| --- | --- | --- | --- | --- | --- |
|  | n = 147 | n=57 | n = 16 | n = 74 |  |
| NYHA class  I  II  III  IV | 46 (45)  35 (34)  9 (9)  13 (13) | 14 (45)  12 (39)  2 (6)  3 (10) | 5 (50)  1 (10)  1(10)  3 (30) | 27 (44)  22 (35)  6 (10)  7 (11) | 0.5147^§^ |
| LVEF | 53 [45, 60] | 54 [46, 62] | 47 [35, 63] | 51 [45, 59] | 0.5606^**^ |
| Troponin T (ng/L) | 19 [10, 39] | 19 [11, 32] | 26 [19, 81] | 14 [10, 40] | 0.2949^**^ |
| NT-pro BNP (pg/ml) | 483 [187, 1092] | 392 [127, 791] | 1014 [187, 3559] | 501 [222, 1294] | 0.4138^**^ |
| Creatinine (µmol/L) | 81 [74, 93] | 81 [74, 102] | 83 [74, 131] | 79 [74, 85] | 0.5232^**^ |

| NYHA, New York Heart Association; LVEF, left ventricular ejection fraction; BNP, brain natriuretic peptide  ^**^ Kruskal-Wallis test  ^§^ Fisher’s exact test |
| --- |

| Supplementary Table 4: Analysis of association of anxiety of COVID-19 and patient characteristics | | |
| --- | --- | --- |
|  | Correlation coefficient | p value |
| CSS |  |  |
| Age | 0.06 | 0.6373^*^ |
| Sex | -0.20 | 0.1096^§^ |
| NYHA class | 0.02 | 0.8534^*^ |
| Intentionally delayed admission | -0.04 | 0.7511^§^ |
| Troponin T | 0.05 | 0.6685^*^ |
| NT-pro BNP | -0.02 | 0.8639^*^ |
| Laevocardiography | -0.30 | **0.0178**^*^ |
| NYHA class at follow up | 0.00 | 0.9829^*^ |
| LVEF at follow up | 0.03 | 0.7760^*^ |
| NT-pro BNP at follow up | 0.13 | 0.4284^*^ |
| FCS-19V |  |  |
| Age | 0.06 | 0.6187^*^ |
| Sex | -0.04 | 0.7245^§^ |
| NYHA class | 0.08 | 0.4463^*^ |
| Intentionally delayed presentation | 0.10 | 0.4143^§^ |
| Troponin T | -0.01 | 0.9220^*^ |
| NT-pro BNP | -0.15 | 0.2161^*^ |
| Laevocardiography | -0.15 | 0.2339^*^ |
| NYHA class at follow up | 0.00 | 0.9907^*^ |
| LVEF at follow up | 0.14 | 0.3156^*^ |
| NT-pro BNP at follow up | 0.20 | 0.2273^*^ |
| Correlation coefficient for continuous variables is denoted as rho (^*^), and for dichotomous variables as r_pbis_ (^§^)  CSS, COVID-19 Stress Scales; FCV-19S, Fear of COVID-19 Scale; NYHA: New York Heart Association; BNP, brain natriuretic peptide; LVEF: left ventricular ejection fraction | | |

Supplementary Figure 1:


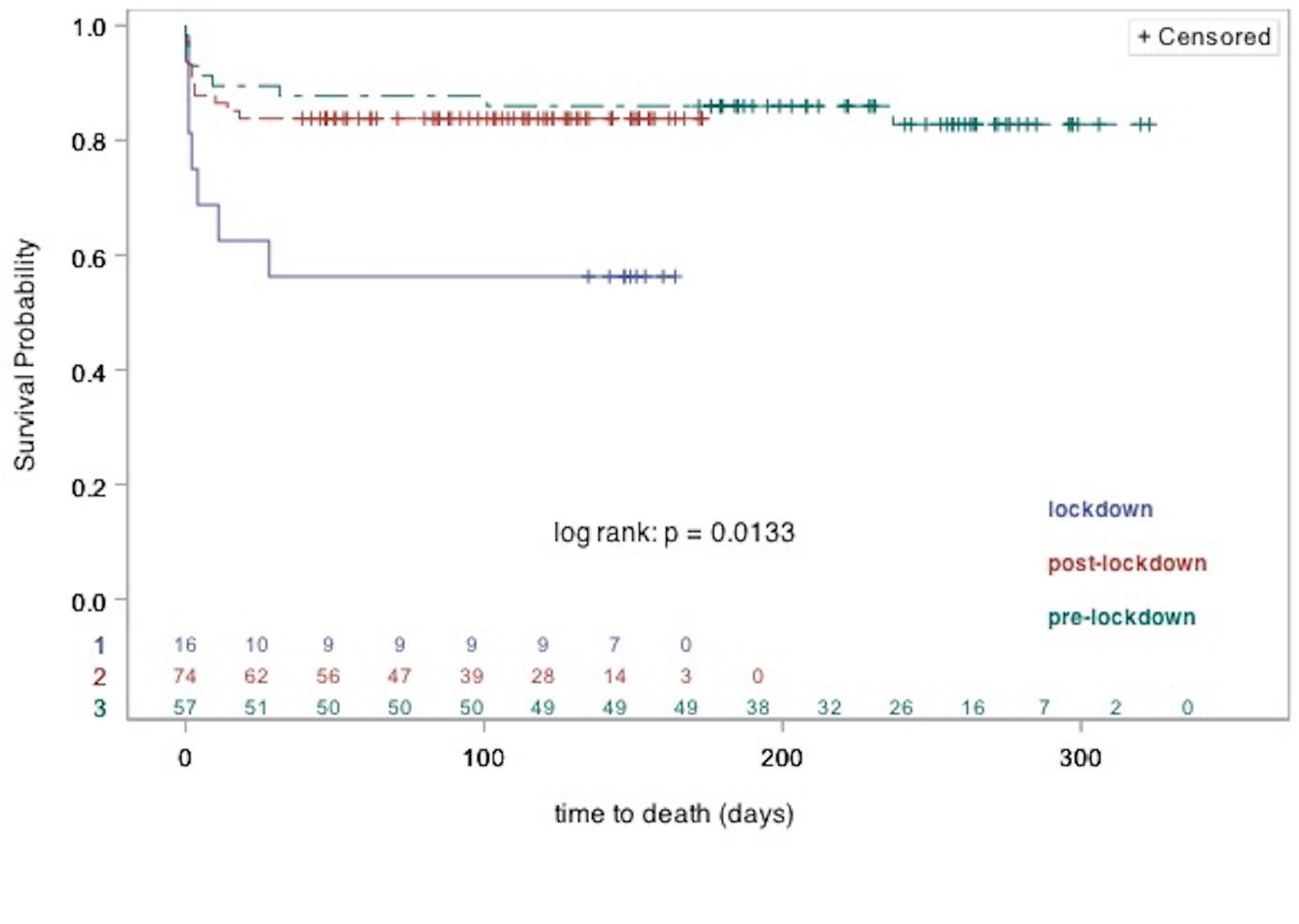
Kaplan Meier plot showing survival of STEMI patients admitted before, during and after COVID-19 associated lockdown.
